# Patient-derived monoclonal LGI1 autoantibodies elicit seizures, behavioral changes and brain MRI abnormalities in rodent models

**DOI:** 10.1016/j.bbi.2025.02.019

**Published:** 2025-02-19

**Authors:** Manoj Upadhya, Alexander Stumpf, Jack O’Brien-Cairney, César Cordero Gómez, Jan Döring, Julius Hoffmann, Susanne Mueller, Yuko Fukata, Scott van Hoof, Divya Dhangar, Max A. Wilson, Arunvir Atwal, Richard Rosch, Gavin Woodhall, Philipp Boehm-Sturm, Masaki Fukata, Jakob Kreye, Dietmar Schmitz, Sukhvir K. Wright, Hans-Christian Kornau, Harald Prüss

**Affiliations:** a Institute of Health and Neurodevelopment, School of Health and Life Sciences, Aston University, Birmingham, United Kingdom; b Charité – Universitätsmedizin Berlin, corporate member of Freie Universität Berlin and Humboldt-Universität zu Berlin, Neuroscience Research Center, Berlin, Germany; c Department of Neurology and Experimental Neurology, Charité – Universitätsmedizin Berlin, corporate member of Freie Universität Berlin and Humboldt-Universität zu Berlin, Berlin, Germany; d German Center for Neurodegenerative Diseases (DZNE) Berlin, Berlin, Germany; e Charité 3R – Replace | Reduce | Refine, Charité – Universitätsmedizin Berlin, corporate member of Freie Universität Berlin and Humboldt-Universität zu Berlin, Berlin, Germany; f Charité Core Facility 7T Experimental MRIs, Charité – Universitätsmedizin Berlin, corporate member of Freie Universität Berlin and Humboldt-Universität zu Berlin, Berlin, Germany; g Division of Molecular and Cellular Pharmacology, Nagoya University Graduate School of Medicine, Nagoya, Japan; h Division of Membrane Physiology, Department of Molecular and Cellular Physiology, National Institute for Physiological Sciences, National Institutes of Natural Sciences, Okazaki, Japan; i Department of Clinical Neurophysiology, King’s College Hospital London NHS Foundation Trust, London, United Kingdom; j Departments of Neurology and Pediatrics, Columbia University, NY, USA; k Division of Neuropharmacology, Nagoya University Graduate School of Medicine, Nagoya, Japan; l Department of Pediatric Neurology, Charité – Universitätsmedizin Berlin, corporate member of Freie Universität Berlin and Humboldt-Universität zu Berlin, Berlin, Germany; m Birmingham Women’s and Children’s Hospital NHS Trust, Birmingham, United Kingdom

**Keywords:** Limbic encephalitis, LGI1, Human monoclonal antibody, Animal model, Seizure

## Abstract

**Objective:**

Limbic encephalitis with leucine-rich glioma inactivated 1 (LGI1) protein autoantibodies is associated with cognitive impairment, psychiatric symptoms, and seizures, including faciobrachial dystonic seizures (FBDS). Patient-derived LGI1-autoantibodies cause isolated symptoms of memory deficits in mice and seizures in rats. Using a multimodal experimental approach, we set out to improve the validity of existing *in vivo* rodent models to further recapitulate the full clinical syndrome of anti-LGI1 antibody mediated disease.

**Methods:**

A monoclonal anti-LGI1 antibody (anti-LGI1 mAb) derived from a patient’s CSF antibody-secreting cell was infused intracerebroventricularly (ICV) into rats and mice for one or two weeks, respectively. Cellular excitability of CA3 pyramidal neurons was determined in hippocampal slices. Structural changes in mouse brains were explored using MRI. Antibody effects on behavior and brain activity of rats were studied using video-EEG.

**Results:**

Anti-LGI1 mAbs augmented the excitability of CA3 pyramidal neurons and elicited convulsive and non-convulsive spontaneous epileptic seizures in mice and rats. Mice displayed a hypoactive and anxious phenotype during behavioral testing. MRI revealed acutely increased hippocampal volume after ICV anti-LGI1 mAb infusion. Video-EEG recordings of juvenile rats uncovered two peaks of seizure frequency during the 7-day antibody infusion period resembling the natural progression of seizures in human anti-LGI1 encephalitis.

**Interpretation:**

Our data strongly corroborate and extend our understanding of the direct pathogenic and epileptogenic role of human LGI1 autoantibodies.

## 1 Introduction

Limbic encephalitis with antibodies to LGI1 is an autoimmune encephalopathy (AIE) that preferentially affects 50-to-70-year-old men. It is characterized by generalized and focal seizures, faciobrachial dystonic seizures (FBDS), spatial disorientation, amnesia and cognitive impairment (Crisp et al., 2016; Dalmau et al., 2017; Dalmau and Graus, 2018; van Sonderen et al., 2017). While one third of patients show a normal MRI in the acute limbic encephalitis stage, hippocampal atrophy and microstructural aberrations in several subfields, including CA1-CA3 areas, dentate gyrus and subiculum, develop in almost all patients in the post-acute phase, paralleled by deficits in verbal and visuospatial memory, attention and executive function (Finke et al., 2017; Miller et al., 2017).

LGI1 is a secreted protein playing crucial roles in the integrity and alignment of pre- and postsynaptic specializations of excitatory synapses (Fukata et al., 2021) as well as in the regulation of intrinsic neuronal excitability (Seagar et al., 2017). Ligand-receptor complexes of LGI1 with ADAM22 and/or ADAM23 are integral to maintaining these functions (Yamagata et al., 2018). Mutations in *LGI1* cause autosomal dominant partial epilepsy with auditory features as well as autosomal dominant lateral temporal lobe epilepsy, and pathogenic variants in the LGI1 receptor *ADAM22* have been associated with infantile-onset refractory epilepsy (van der Knoop et al., 2022). LGI1 knockout mice die from myoclonic epileptic seizures (Chabrol et al., 2010; Fukata et al., 2010; Yu et al., 2010). A study using a series of LGI1 and ADAM22 hypomorphic mice revealed that ~50 % of LGI1 and ~10 % of ADAM22 protein levels are sufficient to prevent lethal epilepsy (Seagar et al., 2017; Yokoi et al., 2021). A recent rat model utilizing the direct inhibition of Kv1.1 currents by dendrotoxin-K (DTX-K) in the primary motor cortex recapitulated the seizures observed in patients with LGI1-Ab encephalitis including FBDS (Baudin et al., 2022). This study high-lights the accumulating evidence that a loss of Kv1 channels that normally dampen down neuronal activity most likely underlies this ictal phenomenon seen in patients (Extrémet et al., 2022; Extrémet et al., 2023; Fukata et al., 2010; Ramirez-Franco et al., 2024; Ritzau-Jost et al., 2024; Seagar et al., 2017).

Previous mouse models based on cerebroventricular transfer of IgG purified from patients’ sera (Petit-Pedrol et al., 2018) show severe impairments of long-term potentiation of the Schaffer collateral pathway and memory deficits in the novel object recognition test. However, spontaneous seizures were not observed. Another recent study using acute and chronic injections of patients’ serum IgG or CSF into the hippocampus and primary motor cortex of mice and rats followed by multisite electrophysiological recordings and video-EEG recordings confirmed the lack of induction of epileptic activity (Baudin et al., 2023). Monoclonal antibodies (mAbs) derived from peripheral B cells of LGI1-Ab encephalitis patients elicited memory deficits, but no ictal activity after injection into hippocampal area CA3 of mice (Ramberger et al., 2020). However, a subset of the same mAbs have produced seizures acutely when infused intracerebroventricularly in juvenile Wistar rats over 7 days, while, interestingly, in this case no cognitive changes were seen (Upadhya et al., 2024).

To gain further insight into direct intrathecal pathogenic effects of autoantibodies in LGI1-Ab encephalitis we infused CSF-cell-derived anti-LGI1 mAbs into the brains of both mice and rats and explored the *in vivo* behavioral, structural, and epileptogenic effects.

## 2 Methods

### LGI1 antibodies

Recombinant human antibodies were purified from cell culture supernatants of HEK293T cells. Antibody-encoding sequences were derived from CSF cells of a patient with a long-lasting progressive anti-LGI1 encephalitis (AIE060), isolated in a previous study (Kornau et al., 2020). For application in animals, we chose AB060-110, an anti-LGI1 antibody that had strongly increased the intrinsic excitability of CA3 pyramidal neurons in hippocampal slice cultures (Kornau et al., 2020) and AB060-154, an LGI1-negative antibody, as a control. The *in vitro* studies additionally included AB060-203 (anti-LGI1) and AB060-144 (control). The control antibodies were expressed from the same vector backbone and derived from the same patient as the anti-LGI1 antibodies but did not bind to LGI1.

### Animals for *in vivo* studies

Twenty postnatal day 21 (P21) male Wistar rats, weighing 50–58 g (Birmingham), and 36 10–12 weeks old C57BL6/J mice (Berlin) were used for the *in vivo* and *in vitro* experiments; one C57BL6/J mouse was subsequently excluded from the experiments because of severe seizure phenotype, in accordance with animal experimentation ethical guidelines. All animals were housed in temperature- and humidity-controlled conditions with a 12 h/12 h light/dark cycle and allowed to feed and drink ad libitum. All procedures involving rats were compliant with current UK Home Office guidelines as required by the Home Office Animals (Scientific Procedures) Act 1986. They were carried out under the authority and procedural approval of a UK Home Office approved project license and in line with ARRIVE guidelines. Local ethical approval for the study was granted by the Aston Bioethics Committee, University of Aston, Birmingham, UK. Studies involving mice were approved by the Landesamt für Gesundheit und Soziales (LaGeSo) in Berlin, Germany (approval number G0078/19), and performed in compliance with German and international guidelines for care and humane use of animals.

Animals used for *in vitro* studies in Japan were reviewed and approved by the ethic committees at the National Institutes of Natural Sciences and were performed according to the institutional guidelines concerning the care and handling of experimental animals. *Adam22*^FAH^ knock-in mice, in which a tandem tag composed of FLAG, AU1 and HA (referred to as an FAH tag) was inserted in the genomic *Adam22* gene (at the extracellular region of ADAM22) were used for experiments for primary cultured neurons (Fukata et al., 2021). They were bred and maintained in the animal facility of the National Institute for Physiological Sciences.

### Mouse experiments

#### Operations and antibody treatment

Experimental animals were randomized for the different treatment groups by an independent investigator. Mice were infused with a total of 25 µg (100 µl, 0.25 µg/µl IgG) of AB060-154 (control) or AB060-110 (anti-LGI1) into the right lateral ventricle over 14 days through continuous intracerebroventricular infusion using surgically implanted osmotic minipumps (model 1002, Alzet, Cupertino, CA). The delivery remained constant from the day of implantation until the end of the experiment. The osmotic pumps delivered at a flow rate of 0.25 µL/hour for a total of 14 days, until the volume loaded in the pump was exhausted.

After surgery, mice were monitored daily to assess symptoms and weight variations. Mice were sacrificed at day 14.

#### Behavioral tests

Behavioral tests were conducted between days 10 and 14 after surgery following established protocols (Jurek et al., 2019; Wolf et al., 2016) All tasks were performed and assessed by trained experimenters under blinded conditions and contained Open Field (locomotion and anxiety), Rotarod (motor function), Y-maze (willingness to explore new environments), and Three-Chamber test (sociability, interaction with unfamiliar mouse). Prior to testing, all animals were given at least 30 min of acclimation to the facility. The tests were recorded using a camera mounted above the maze; TSE Video Mot 3D Classic V8.02 software was used to track and record the animals.

#### MRI measurements

Mice were anesthetized with 1–2 % isoflurane in a 70:30 nitrous oxide:oxygen mixture. Body temperature and respiration rate were monitored with MRI compatible equipment (Small Animal Instruments, Inc., Stony Brook, NY). T2-weighted (T2w) images were acquired 14 days post-surgery on a 7 Tesla rodent scanner (BioSpec 70/20 USR, Bruker, Ettlingen, Germany) with a 1H-Cryo Probe (Bruker). A 2D turbo spin echo pulse sequence was used with 40 contiguous slices covering the whole brain including olfactory bulb and cerebellum, 0.40 mm slice thickness, field of view of 19.2 x 19.2 mm^2^, image matrix of 192 x 192, repetition time/effective echo time = 4250 ms/33 ms, RARE factor = 8, number of averages = 2 and a total acquisition time of 3:26 min.

#### Electrophysiology

Hippocampal slices were prepared from mice after 14 days of antibody treatment and whole cell patch clamp recordings were performed to assess the excitability of the cells as previously described (Kuijpers et al., 2021; Stempel et al., 2016). For input–output curves, the number of APs was plotted with the injected current. The rheobase represents the minimal current eliciting at least 1 AP; input resistance was detected in current clamp configuration by measuring the voltage deflection of a hyperpolarizing current pulse. The inter-spike-interval (ISI) represents the time between two APs and was measured at current injection step where the most APs occurred. ISI-ratio is calculated as a quotient of the shortest ISI of a cell divided by the average ISI of the same cell^26^ and can be used to quantify bursting. DTX-K (100 nM) was acutely washed-in to the slices and applied for at least 10 min before measurements.

#### Immunoprecipitation and western blot analysis of mouse primary neuron culture

Hippocampal and cortical neurons were dissociated from brains of E16 homozygous *Adam22*^FAH^ knock-in mice and seeded onto 0.1 % polyethylenimine and 50 μg/ml collagen I–coated 12-mm coverslips (24-well plate) in Neurobasal medium with 2 mM GlutaMax, B-27 and 9 % fetal bovine serum. After 3-hour incubation, the media was changed to Neurobasal medium supplemented with 2 mM GlutaMax and B-27. To examine the effect of LGI1 antibodies in neurons, neurons (~25 DIV) were treated with purified antibody clones for 2–3 days. Purified antibody IgG (1 mg/ml in PBS) was added at 5 µg/ml in the culture medium and supplemented once 24 h later at 2.5 µg/ml (final 7.5 µg/ml concentration). AB060-144 or AB060-154 was used as the control IgG. For immunoprecipitation assay, cultured neurons were extracted with buffer A[20 mM Tris-HCl (pH 8.0), 1 mM EDTA, 100 mM NaCl, 1 % Triton X-100 and 50 μg/ml PMSF]. The lysates were cleared by centrifugation at 10,000 g for 5 min at 4 °C. ADAM22-FAH was precipitated with FLAG-M2 agarose (Sigma-Aldrich) for 1 h, washed with buffer A, and eluted with buffer A containing 0.25 mg/ml FLAG peptide. The eluates were subjected to SDS-PAGE and analyzed by western blotting with rabbit monoclonal antibody to HA (for ADAM22-FAH; clone C29F4, Cell Signaling Technology #3724), guinea pig polyclonal antibody to LGI1 (Nittobo, LGI1-GP-Af510), and mouse monoclonal antibodies to PSD-95 (clone K28/43, NeuroMab #75–028) and to Kv1.2 (clone K14/16, NeuroMab #75–008). For western blotting, chemical luminescent signals were detected with a cooled CCD camera and the band intensities were analyzed (FUSION Solo system, Vilber-Lourmat).

Statistical details of individual experiments are described in figure legends. To perform statistical analysis, at least three independent biological samples from 2 to 3 different animal pairs were included in the analyses. For multiple test subjects, Kruskal-Wallis with post-hoc Scheffe test (non-parametric) or one-way repeated measures ANOVA with post-hoc Bonferroni test (parametric) was used. Results are shown as means ± SE. Statistical analysis was performed with Ekuseru-Toukei 2012 software (BellCurve).

### Rat experiments

#### Operations and antibody treatment

Rats were implanted with osmotic pumps and transmitters for *in vivo* EEG recordings as previously reported (Kreye et al., 2021a; Upadhya et al., 2024; Wright et al., 2021), Briefly, a total of 70 µg of control antibody (AB060-154) or anti-LGI1 (AB060-110) was infused by cerebroventricular route using bilateral osmotic pumps (model 1007D, Azlet) (volume 100 µl, flow rate 0.5 µl, duration 7 days), primed over-night, connected to intraventricular cannulae (328OPD-3.0/SPC; PlasticsOne; implanted into lateral ventricles −1.5 mm lateral, 0.6 mm caudal) via polyethylene tubing 69 mm-×-1.14 mm diameter (C312VT; PlasticsOne). The EEG transmitter was placed subcutaneously (A3028B-DD subcutaneous transmitters, 90-mm leads, OpenSource Instruments (OSI)), and depth electrode (W-Electrode (SCE-W), OSI) placed in the left hippocampus (CA3, 3.5 mm lateral, 3.6 mm caudal, depth 2.3 mm) with a reference electrode implanted on the contralateral skull surface (3.5 mm lateral, 3.6 mm caudal), and secured. For 48 h *in vitro* electrophysiology experiments a single dose (10 µl) of anti-LGI1 (AB060-110) or control antibody (AB060-154) (10 µl) was injected ICV into left lateral ventricle (1.5 mm left lateral and 0.6 mm caudal from bregma) (Wright et al., 2021).

#### Video EEG recordings

The EEG recordings and videos (OSI) were collected using our standardized laboratory protocol (Kreye et al., 2021a; Upadhya et al., 2024; Wright et al., 2015; Wright et al., 2021). In brief, EEG data (wide band pass 0.2–160 Hz sampled at 512 samples per second) was collected and recorded using Neuroarchiver software (OSI) from freely moving animals placed in a custom-built Faraday cage. Video archiver software and Animal Cage Cameras (https://www.opensourceinstruments.com/Electronics/A3034/M3034.html), provided simultaneous and synchronous video alongside EEG signals. Video-EEG matching was used to identify ictal EEG events and Event Classifier (OSI) used to classify one-second segments of EEG according to program metrics (power, coastline, intermittency, coherence, asymmetry, spikiness), generating a library of ictal events allowing identification of abnormal EEG events by automated comparison to the library. To identify seizures the Seizure Consolidation Tool (CEL4V1.tcl, OSI) which scans the EEG file for strings of consecutive ictal events above a threshold length was used. Seizures were considered separate events if the duration between neighboring ictal events was longer than a threshold value (max_-break_intervals). The minimum number of start intervals was set to 4, the maximum number of break intervals was set to 10 (default), and the minimum seizure length was set to 8 s (length of a playback interval). The powerband analysis was carried out using a custom-designed macro. Statistical analysis was conducted using Graphpad Prism 8 (GraphPad Software Inc.). Video-EEG analysis of seizure phenotypes was carried out by two independent observers blinded to treatment group.

#### Behavioral tests

The Post-Seizure Behavioral Battery (PSBB) was performed as previously described to assess hyperexcitability and other behavioural indices suggestive of epilepsy (Kreye et al., 2021b; Modebadze et al., 2016; Upadhya et al., 2024). Two simple and non-stressful tasks constituted the PSBB (touch and pick up task). PSBB scores were calculated by taking the product of the touch and pickup scores (‘touch x pickup’), a score > 10 indicated hyperexcitability.

#### Electrophysiology

As described previously (Wright et al., 2021), for whole-cell patch clamp recordings, rat brain slices were prepared at a thickness of 350 µm. For spike/current intensity curves, the number of action potentials was plotted against the injected current. Input resistance was measured from the slope of a linear regression fit to the voltage-current plotted graph between −80pA to 0pA. Data were analyzed using Axograph and Prism 8, statistical tests used to compare groups (Mann–Whitney and Wilcoxon matched pairs ranked tests) were one-tailed. Measurements are expressed as mean ± SEM.

#### Immunofluorescence and image analysis

Immunofluorescence studies were performed following *in vitro* electrophysiology experiments on 350 µm brain slices and images analyzed as previously described (Upadhya et al., 2024; Wright et al., 2021). Statistical analysis was performed using GraphPad Prism 8 software.

## 3 Results

### Human anti-LGI1 mAbs cause behavioral abnormalities, seizures and MRI changes *in vivo* and hyperexcitability *in vitro* when infused into mice

**Fig. 1:**
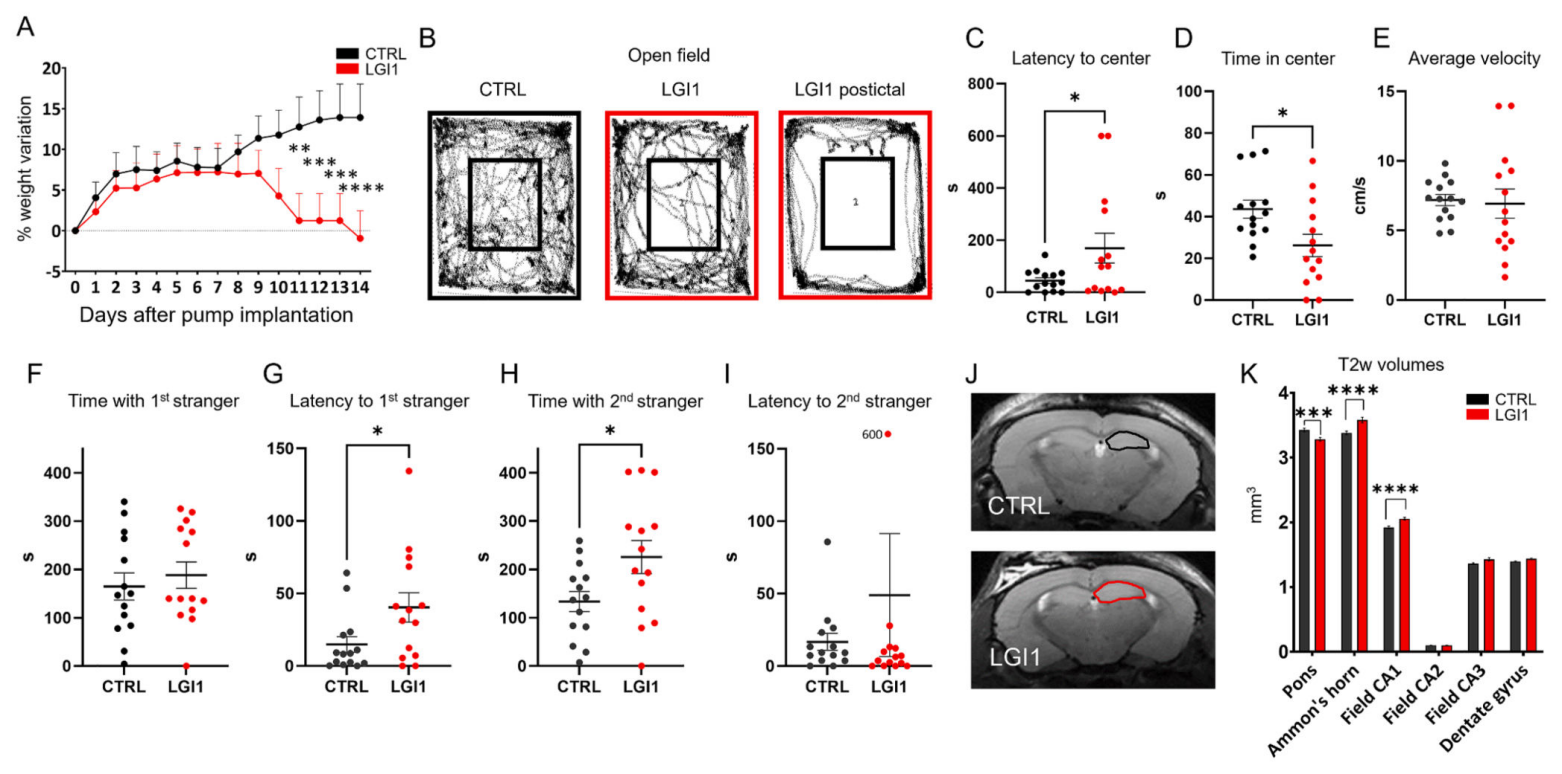
Behavioral effects and MRI changes following intrathecal infusion of monoclonal human LGI1 autoantibodies into mice. (A) Marked loss of body weight in mice receiving intracerebroventricular administration of anti-LGI1 antibodies compared to controls (mean ± standard error of the mean [SEM] from 14 animals per group from days 0 to 14 [multiple *t*-test comparisons: *p < 0.05, **p < 0.01, ***p < 0.001, ****p < 0.0001]). (B) Representative recordings of Open Field test tracks from mice receiving control (left) or anti-LGI1 mAbs (middle). In individual mice, seizures led to reduced post-ictal motility (right). (C, D) Increased latency to visit the center area of the open field (C, p = 0.0413) and reduced mean time in the center area (D, p = 0.0178) in anti-LGI1 mAb treated compared to control mice, indicating anxiety-like behavior. (E) Unchanged mean velocity indicates normal (loco)motor function. (F, G) In the three-chamber test, mice of both groups spent the same time with a first stranger mouse (F), but latency to make contact was markedly increased in anti-LGI1 mAb-treated animals, indicating potential deficits in sociability (p = 0.0358, G). (H, I) Introduction of a second stranger mouse led to increased investigation by LGI1 mAb-infused mice, indicating increased interest in social novelty (p = 0.0300, H). Latency to make contact with the novel intruder was unchanged; one mouse did not move and therefore gave the high value (600 s) in the LGI1 group (I). (J, K) Representative T2w anatomical MR images (n = 13 animals per group; 40 slices per brain) of mice treated with either control or anti-LGI1 mAbs (J). Quantification showed significantly increased hippocampal volumes and subtly reduced pontine volumes after treatment with anti-LGI1 mAbs (K, *t*-test comparisons; FDR correction = 0.1; significance levels *p < 0.05, **p < 0.01, ***p < 0.001, ****p < 0.0001).

Previous studies have shown that infusion of anti-LGI1 antibodies into mouse brains lead to memory deficits, but not seizures (Petit-Pedrol et al., 2018; Ramberger et al., 2020) one of the core symptoms of limbic encephalitis with LGI antibodies. We set out to test the *in vivo* effects of an anti-LGI1 mAb that strongly increased the intrinsic excitability of CA3 pyramidal neurons in hippocampal slice cultures (Kornau et al., 2020). Mice were infused with a dose of 25 µg into the right cerebral ventricle over 14 days and assessed daily for body weight changes and presence of neurological symptoms. Weight loss was seen in those receiving anti-LGI1 mAbs compared to control antibody injected mice from day 7 (Fig. 1A). In animals treated with anti-LGI1 mAbs we observed overt epileptic seizures and phases of nearly complete akinesia possibly suggesting postictal states, which likely interfered with food intake.

Behavioral tests revealed that animals receiving anti-LGI1 mAbs showed an increased latency to enter the center of the open field as compared to the control group (p = 0.0413, Fig. 1B-C). The mean time in the center was significantly reduced in LGI1 mice (p = 0.0178, Fig. 1D), in particular in 3/14 mice which had seizures during the tests with markedly reduced center time (Fig. 1B, right). Avoidance of the center area was not related to impaired locomotion as distance travelled and mean velocity were unchanged (Fig. 1E). No differences between groups could be detected in rotarod and Y-maze tests (data not shown). Mice of both groups spent the same time with a first stranger mouse in the three-chamber test (Fig. 1F), but latency to make contact with the stranger mouse was markedly increased in anti-LGI1 mAb-treated animals (p = 0.0358, Fig. 1G), indicating potential deficits in sociability. After the introduction of a second stranger mouse, LGI1 animals showed increased investigation of the novel intruder compared to control-treated mice, indicating increased interest in social novelty (p = 0.0300, Fig. 1H-I). These results indicate a causal link between anti-LGI1 mAbs and epileptic seizures and suggest that the autoantibodies may promote behavioral abnormalities including anxiety-like behavior and dysfunctional social behavior.

We then assessed pathological hallmarks induced by the administration of anti-LGI1 mAbs using MRI. T2 weighted (T2w) anatomical images in mice did not reveal focal antibody-driven lesions among the different treatment groups. Significant differences were observed in both the hippocampal formation and the pons (Fig. 1 J, K). In theAmmońs horn, anti-LGI1 mAb infused animals exhibited larger T2w-volumes compared to control mAB treated mice. Specifically, larger volumes were identified in the CA1 region, but not in the CA2 and CA3 (p = 0.0001). Conversely, a reduction in T2w-volume was observed in the pons in anti-LGI1 mAb infused animals compared to control mAb treated mice (p = 0.001), suggesting region-specific differential effects of anti-LGI1 mAb. All further analysed anatomical areas did not show differences among the treatment groups (Fig. 1K).

**Fig. 2:**
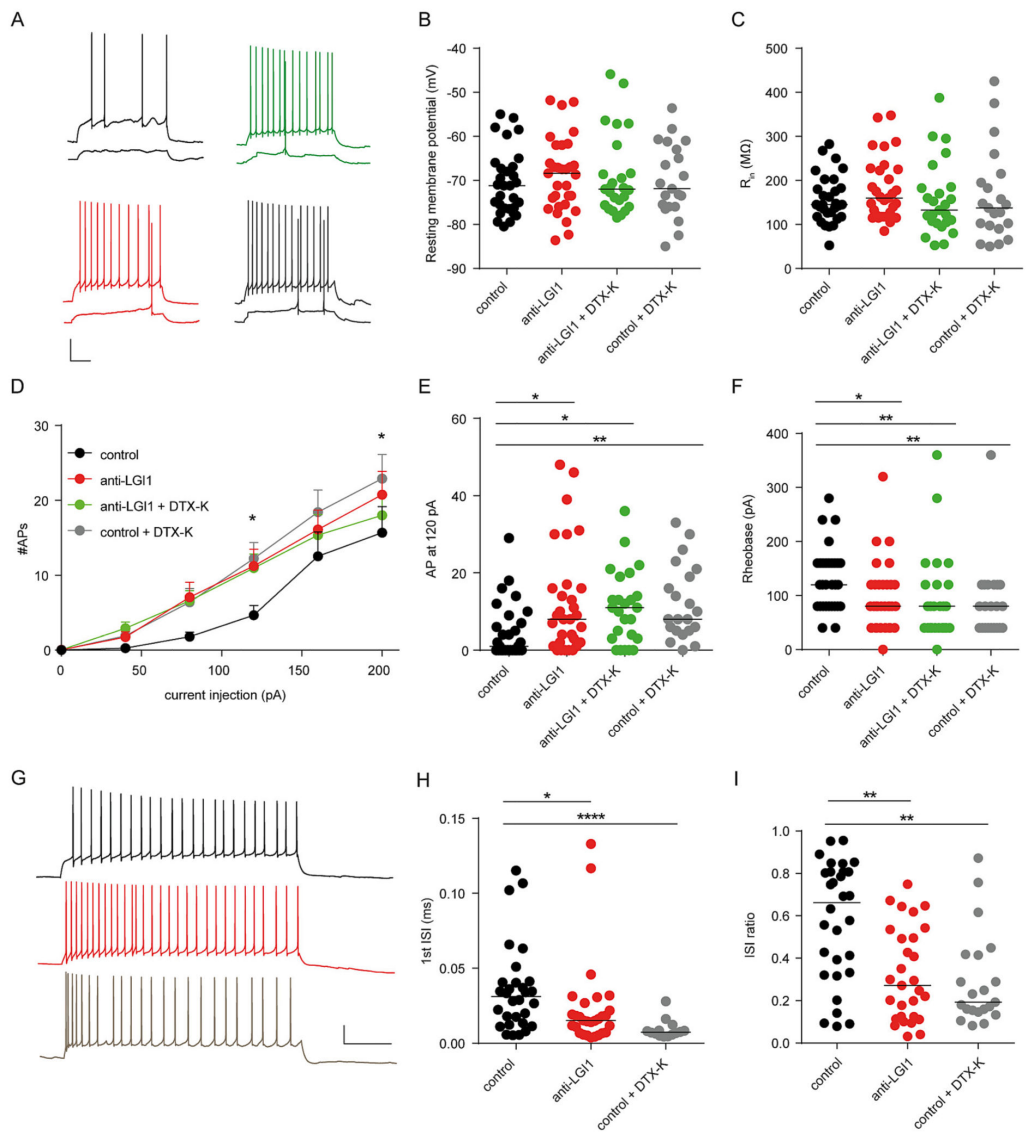
*In vivo* treatment with anti-LGI1 mAbs in mice increases excitability and bursting of CA3 pyramidal neurons. (A) Example traces of cell responses during depolarizing current steps (40pA and 120pA). Black: control; red: slices from anti-LGI1 mAb treated mice; green: slices from anti-LGI1 mAb treated mice after DTX-K (100 nM) wash-in; gray: slices from control mice after DTX-K wash-in. Scale bar: 20 mV/0.2 s. (B, C) Resting membrane potential (B, ANOVA (Kruskal-Wallis test): *p* = 0.8251) and input resistance (C, ANOVA (Kruskal-Wallis-Test): *p* = 0.3968) were not altered with LGI or DTX-K treatment. (D) AP number/current intensity relationship displays a significant increase in excitability when mice were perfused anti-LGI1 mAb or slices treated with DTX-K. Data points represent mean ± SEM. 2-way-ANOVA, Dunnet’s multiple comparison test: 120pA: control vs LGI * (CI −12.82 to −0.3107) control vs control DTX * (CI = −14.47 to −0.6777); 200pA: control vs. control DTX * (CI: −14.10 to −0.3111). (E) The number of spikes elicited by 120pA current injection was significant higher when mice were perfused with anti-LGI1 mAb or slices treated with DTX-K: ANOVA (Kruskal-Wallis-Test): p = 0.0027; Dunnet’s multiple comparison Test: *p < 0.05; **p < 0.01.

In line with the effects of anti-LGI1 mAbs in slice cultures (Kornau et al., 2020), we detected an increased intrinsic excitability and bursting in CA3 pyramidal neurons in acute hippocampal slices after two weeks of antibody treatment (Fig. 2A). While resting membrane potential and input resistance did not change significantly after treatment with anti-LGI1 mAbs, DTX-K or both, compared to control antibodies (Fig. 2B, C), the treated cells showed an increased firing with increased current injection (Fig. 2D, E) and a reduced rheobase (Fig. 2F). In addition, the first inter-spike-interval (ISI) as well as the ISI-ratio were reduced in cells treated with anti-LGI1 mAbs and this effect was mimicked by application of DTX-K to control cells (Fig. 2G, H, I), indicating increased bursting behavior after treatment with anti-LGI1 mAbs.

(F) The rheobase was significant higher when mice were perfused with anti-LGI1 mAb or slices treated with DTX-K: ANOVA (Kruskal-Wallis-Test): *p* = 0.0052; Dunnet’s multiple comparison Test: *p < 0.05; **p < 0.01. (G) Example traces of cell responses during depolarizing current steps eliciting the maximal number of spikes. Scale bar: 20 mV/0.2 s. (H) The duration of the first inter-spike interval (ISI) was reduced in cells from anti-LGI1 mAb perfused mice and in DTX-K treated slices: ANOVA (Kruskal-Wallis-Test): *p* < 0.0001; Dunnet’s multiple comparison Test; *p < 0.05; ****p < 0.0001. (I) The inter-spike interval (ISI) ratio (ISI_MIN_ / ISI_average_) was reduced in cells from anti-LGI1 mAb perfused mice and in DTX-K treated slices; ANOVA (Kruskal-Wallis-Test): *p* = 0.0004; Dunnet’s multiple comparison Test; **p < 0.01.

Graphs show single datapoints (cells) with the median unless otherwise stated. For each condition, 4 mice were used. Number of cells in B, C, E, F: control, n = 30; LGI, n = 31, LGI + DTX, n = 25; control + DTX, n = 21; in D: control, n = 30; LGI, n = 30, LGI + DTX, n = 25; control + DTX, n = 21; in H and I: control, n = 30; LGI, n = 29; control + DTX, n = 21.

### Seizures in human-derived anti-LGI1 mAbs infused juvenile Wistar rats recapitulate the patient seizure phenotype

**Fig. 3:**
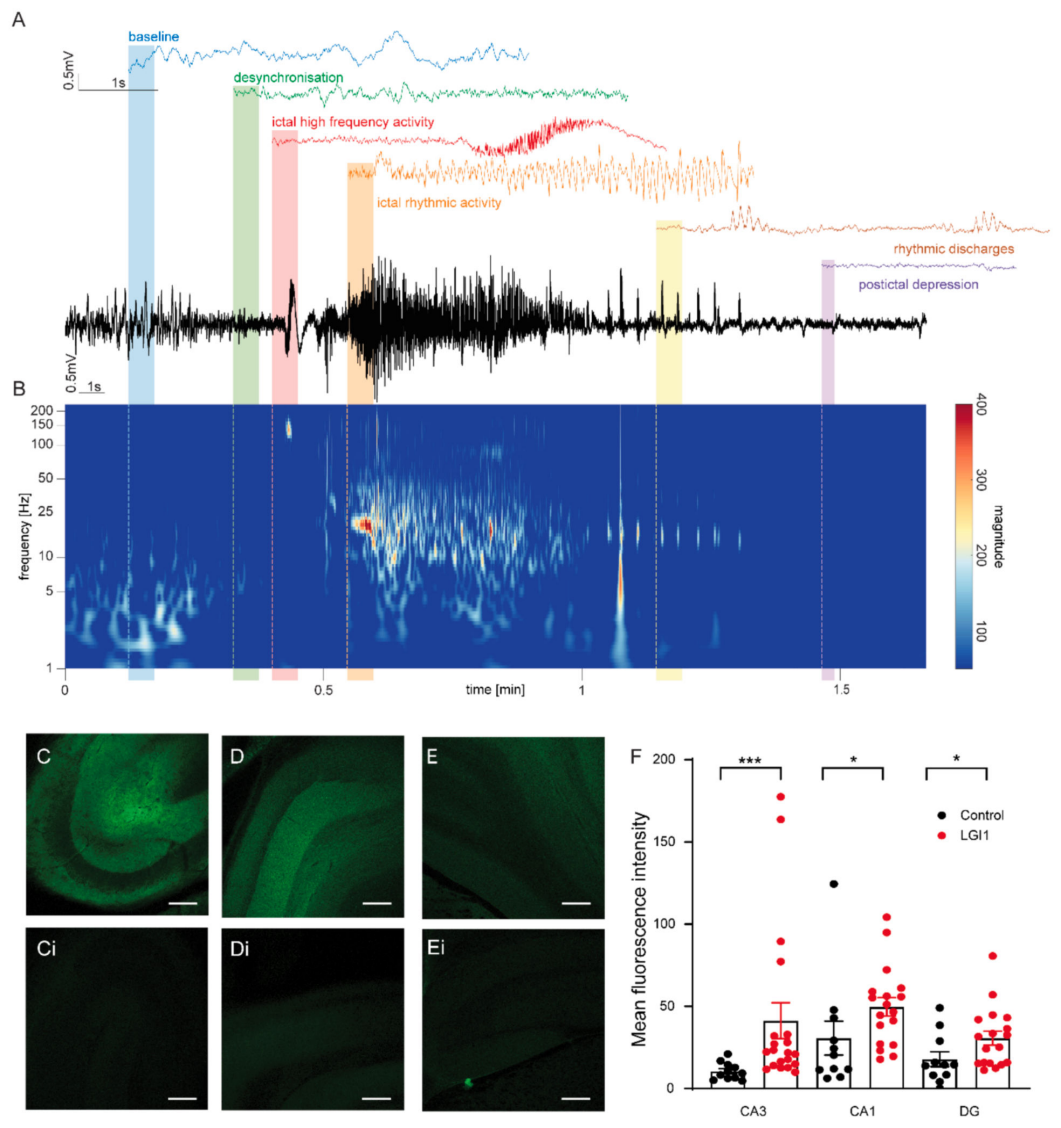
Electroclinical features of anti-LGI1 mAb rat seizure model. (A) Example EEG trace (black) from anti-LGI1 mAb infused rat recorded from depth electrode placed in CA3 region of hippocampus with highlighted, color-coded breakdown of seizure EEG features at different phases of the seizure. (B) Temporally aligned time–frequency plot, illustrating the spectral evolution of the EEG signal. (C) Confocal image of CA3 region of hippocampus from sagittal brain slice prepared 14 days after 7-day infusion of anti-LGI1 mAbs with accompanying image of CA3 in control mAb infused brain slice (Ci). Scale bar 250 µm. (D) Confocal image of CA1 region of hippocampus from sagittal brain slice prepared 14 days after 7 day infusion of anti-LGI1 mAbs with accompanying image of CA3 in control mAb infused brain slice (Di). Scale bar 250 µm. (E) Confocal image of dentate gyrus (DG) of hippocampus from sagittal brain slice prepared 14 days after 7-day infusion of anti-LGI1 mAbs with accompanying image of CA3 in control mAb infused brain slice (Ei). Scale bar 250 µm. (F) Mean fluorescence intensity of CA3, CA1 and DG of brain slices infused for 7 days with anti-LGI1 or control mAb. Data is expressed as mean ± SEM of anti-LGI1 mAb in CA3 and CA1 (n = 20) and anti-LGI1 mAb in DG (n = 19) and for control mAb in CA3, CA1 and DG (n = 11). (***p < 0.001, *p < 0.05; Mann Whitney *t*-test). Measurements shown as mean ± SEM. Green fluorescence staining represents human LGI1-antibody binding.

Our experiments in mice indicated a direct causal relation between anti-LGI1 mAbs (AB060-110), hyperexcitability and seizures. To explore the epileptogenicity of anti-LGI1 mAbs more closely, we implanted 7-day osmotic pumps to deliver control (n = 5) or anti-LGI1 mAbs (n = 6) into the lateral cerebral ventricles of juvenile Wistar rats with wireless EEG transmitters recording from a depth electrode placed in hippocampal CA3. Continuous EEG was recorded for up to 21 days (battery life of transmitter) in the freely moving rats, after which the rats were culled. Spontaneous epileptic seizures were observed in all 6 anti-LGI1 mAb infused rats and none of the 5 controls (one control EEG recording was not suitable for analysis due to post-implantation disconnection of reference electrode recording wire). The electroclinical features of the seizures were highly stereotyped (Fig. 3A,B). Convulsive seizures were characterized by behavioral arrest, unilateral tonic paw extension (seen in 29 % of seizures analyzed; 24/83) with upward tilting of the head which then progressed to rearing before full tonic-clonic seizure movements (illustrated in Fig. 3C and Supplementary video 1). Binding of the anti-LGI1 mAbs was confirmed on postmortem immunohistochemistry (see Fig. 3D-G).

**Fig. 4:**
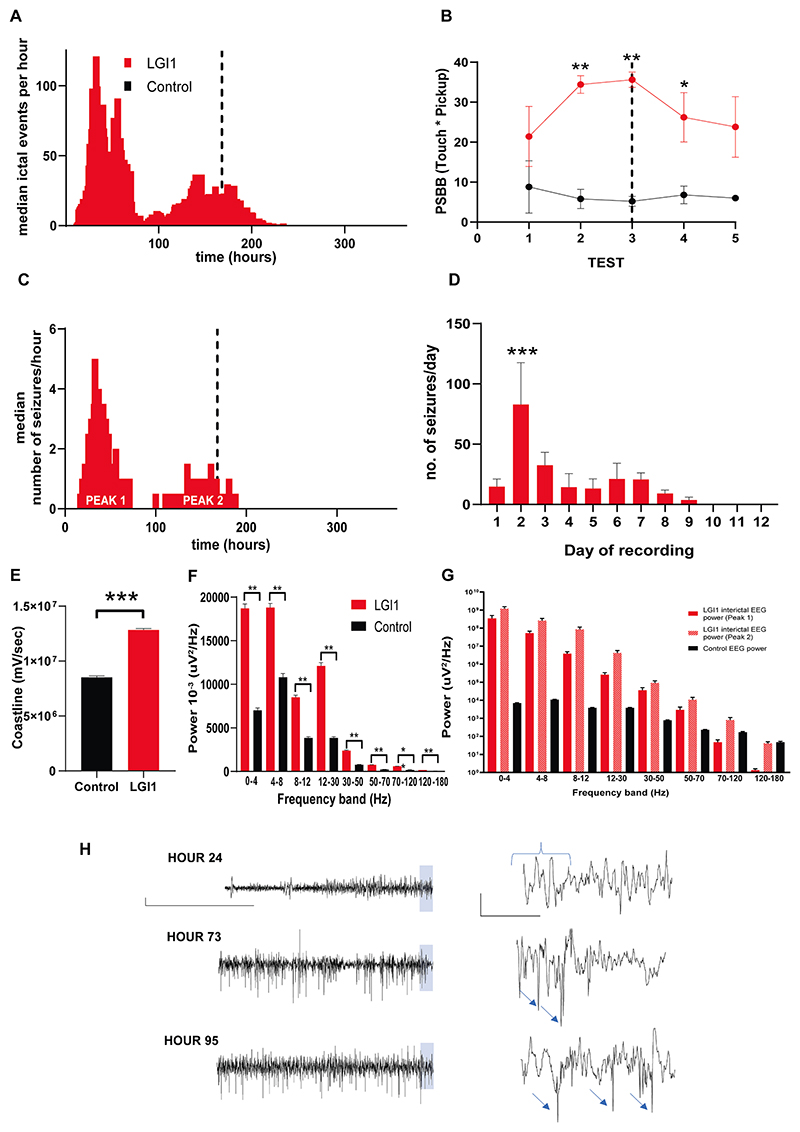
EEG of anti-LGI1 mAb seizures in rats show a bimodal peak during antibody infusion and deterioration of interictal/baseline EEG over time. (A) The median hourly number of 1-second ictal EEG events per hour in anti-LGI1 mAb-infused rats over time (n = 6) as detected by automated seizure detection software (Opensource Instruments). No ictal events were seen in the control rats(n = 5). (B) PSBB scores as measures of hyperexcitable behavior in anti-LGI1 mAb-infused rats (n = 6) as compared to controls (n = 5) using the PSBB (post-seizure behavioral battery) test (**p < 0.001; 2-way ANOVA with Bonferroni multiple comparisons test). The dotted line in both graphs represents the end of the 7-day antibody infusion. Measurements shown as median or mean ± SEM. (C) Median number of seizures per hour (concatenating individual ictal events) over the duration of antibody infusion (dotted line represents end of antibody infusion). (D) Daily comparison of seizure frequency (***p < 0.001; Ordinary one-way ANOVA). Measurements shown as mean ± SEM. (E) EEG coastline length (calculated per hour for entire 21-day recording period for each animal) in anti-LGI1 mAb-infused (n = 6) and control mAb-infused animals (n = 5) (***p < 0.001; Mann-Whitney). Measurements shown as median or mean ± SEM. (F) EEG power in different frequency bands (calculated per hour for 21 day recording period for each animal) in anti-LGI1 mAb infused (n = 6) and control mAb infused animals (n = 5) (**p < 0.01; Mann-Whitney). Measurements shown as mean ± SEM. (G) Comparison of frequency-band specific EEG power during interictal segments recorded during periods with peak seizure frequencies – Peak 1 (red) and Peak 2 (hashed red) in anti-LGI mAb infused animals; compared to controls (black). Measurements shown as mean ± SEM and expressed in log scale. (H) Example of interictal EEG in anti-LGI mAb infused animal at 24, 73 and 95 h. Blue highlighted panel on left panel EEGs shown enlarged in corresponding right panel EEG trace. Blue bracket indicates normal sleep EEG pattern. Blue arrows indicate interictal spikes. Scale bar for left panel 1 mV vs 50 s and for right panel 1 mV vs 5 s.

Using automated EEG seizure detection, a significant number of ictal events per hour were seen during and shortly after the 7-day infusion (Fig. 4A). A behavioral test of hyperexcitability, the post seizure behavioral battery (PSBB), showed rats infused with anti-LGI1 mAbs had significantly higher scores than controls (high score > 10) (Fig. 4B) throughout the infusion period and the week after. PSBB scores are consistently raised in rats with spontaneous recurrent seizures(Kreye et al., 2021a; Modebadze et al., 2016) Using seizure consolidator software adjacent the 1 s ictal events were concatenated to identify seizures of varying duration. These showed two peaks of seizure activity, with the highest number of seizures seen on Day 2 of infusion/recording (Fig. 4C, D). Seizure length did not differ significantly between Peak 1 (n = 59) and 2 (n = 27) seizures (47.8 s ± 3.8 s vs 56.7 s ± 6.1 s; p = ns, Mann-Whitney). Video-EEG was used to examine 402 seizures from 5 anti-LGI1 mAb infused rats, 76 % (304/402) were clearly visible to categorize into non-convulsive 54 % (219/402) and convulsive 21 % (85/402). The proportion of non-convulsive seizures increased in Peak 2 (91 % vs. 70 % in Peak 1). By Day 10, three days after completion of the antibody infusion, seizures had resolved (Fig. 4D).

A significant increase in the EEG coastline (a measure sensitive to differences in fast frequency and high amplitude EEG content) of the anti-LGI1 mAb infused rats compared to the control mAb infused rats was expected secondary to the increased number of seizures (Fig. 4E). The EEG of the anti-LGI1 mAb infused animals showed a significantly increased power in all the frequency wavebands (Kreye et al., 2021a; Wright et al., 2021) (Fig. 4F). Further observation and analysis of the baseline EEG in anti-LGI1 mAb-infused rats revealed increasing interictal spike activity most likely contributing to the increased coastline and frequency band power (Fig. 4G). As the anti-LGI1 mAb infusion time increased, the EEG showed an increasing number of interictal spikes (Fig. 4H).

### Anti-LGI1 mAbs cause increased intrinsic cell excitability *in vitro* 48 h after intracerebroventricular (ICV) injection in juvenile Wistar rats

**Fig. 5:**
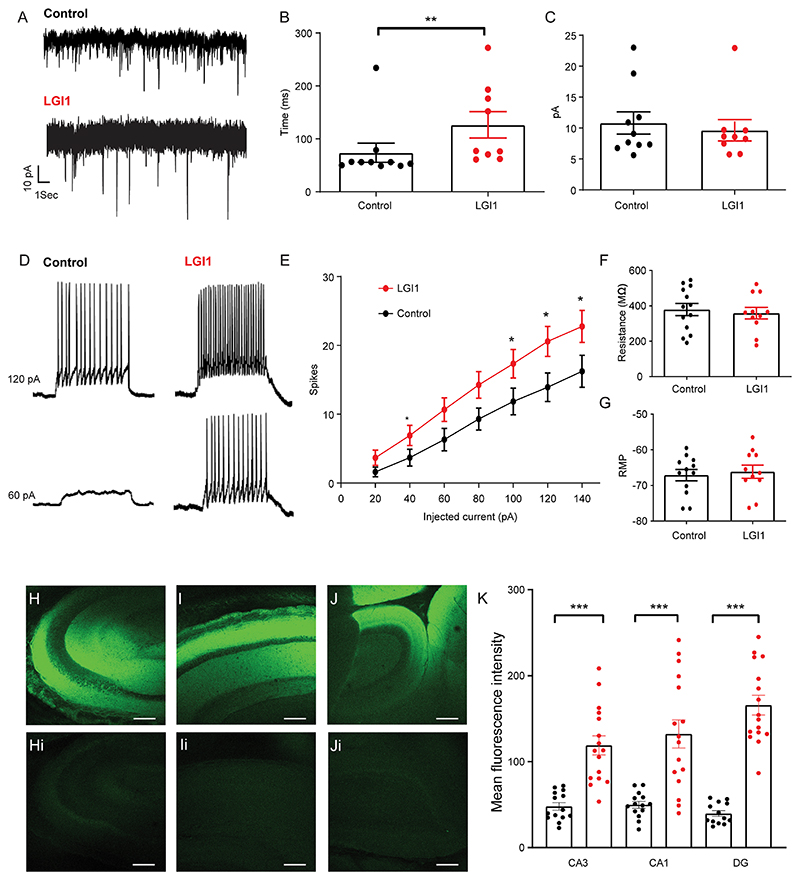
Electrophysiological recordings *in vitro* 48 h after single intracerebroventricular (ICV) injection of anti-LGI mAbs into rats show increased excitability in CA3 pyramidal neurons. (A) Representative spontaneous EPSC (sEPSC) whole-cell patch clamp recording 48 h after ICV injection with anti-LGI 1(lower trace) or control mAbs (upper trace). Scale bar 10pA vs 1 s. (B) The interevent interval of the sEPSCs recorded from CA3 pyramidal cells in control (n = 10 cells from 4 rats) and anti-LGI1 mAb (n = 9 cells from 5 rats) injected slices 48 h after ICV injection (**p = 0.008; Mann-Whitney). Measurements shown as mean ± SEM.(C) The amplitude of the sEPSCs recorded from CA3 pyramidal cells in control (n = 10 cells from 4 rats) and anti-LGI1 mAb (n = 9 cells from 5 rats) injected slices 48 h after ICV injection (*p* = ns; Mann-Whitney). Measurements shown as mean ± SEM. (D) Representative traces of CA3 pyramidal cell responses during depolarizing current steps in CA3 pyramidal cells from control (left traces) and anti-LGI1-mAb (right traces) injected rodent slices 48 h after ICV injection. (E) Depolarizing steps of different current intensities elicited significantly more spikes in the anti-LGI1 mAb injected rodent slices (n = 11 cells from 3 rats) than in control antibody (n = 13 cells from 4 rats) treated slices at 40, 100, 120 and 140pA currents (*p < 0.05, unpaired *t*-test). Measurements shown as mean ± SEM. (F) Input resistance was not significantly altered between the two conditions (*p* = ns; Mann-Whitney). Measurements shown as mean ± standard error of the mean (SEM). (G) The resting membrane potential was not significantly different between the two conditions (*p* = ns; Mann-Whitney). Measurements shown as mean ± standard error of the mean (SEM). (H) Confocal image of CA3 region of hippocampus from sagittal brain slice prepared 48hrs after ICV injection of anti-LGI1 mAbs with accompanying image of CA3 in control mAb infused brain slice (Hi). Scale bar 250 µm. (I) Confocal image of CA1 region of hippocampus from sagittal brain slice prepared 48hrs after ICV injection of anti-LGI1 mAbs with accompanying image of CA1 in control mAb infused brain slice (Ii). Scale bar 250 µm. (J) Confocal image of dentate gyrus (DG) of hippocampus from sagittal brain slice prepared 48hrs after ICV injection of anti-LGI1 mAbs with accompanying image of DG in control mAb infused brain slice (Ji). Scale bar 250 µm. (K) Mean fluorescence intensity of CA3, CA1 and DG of brain slices injected with single ICV dose of anti-LGI1 mAb or control mAb. Data is expressed as mean ± SEM of anti-LGI1 mAb in CA3, CA1 and DG (n = 16) and for control mAb in CA3 and CA1 (n = 14) and in DG (n = 13). (***p < 0.001; Mann Whitney *t*-test). Measurements shown as mean ± SEM.

**Fig. 6:**
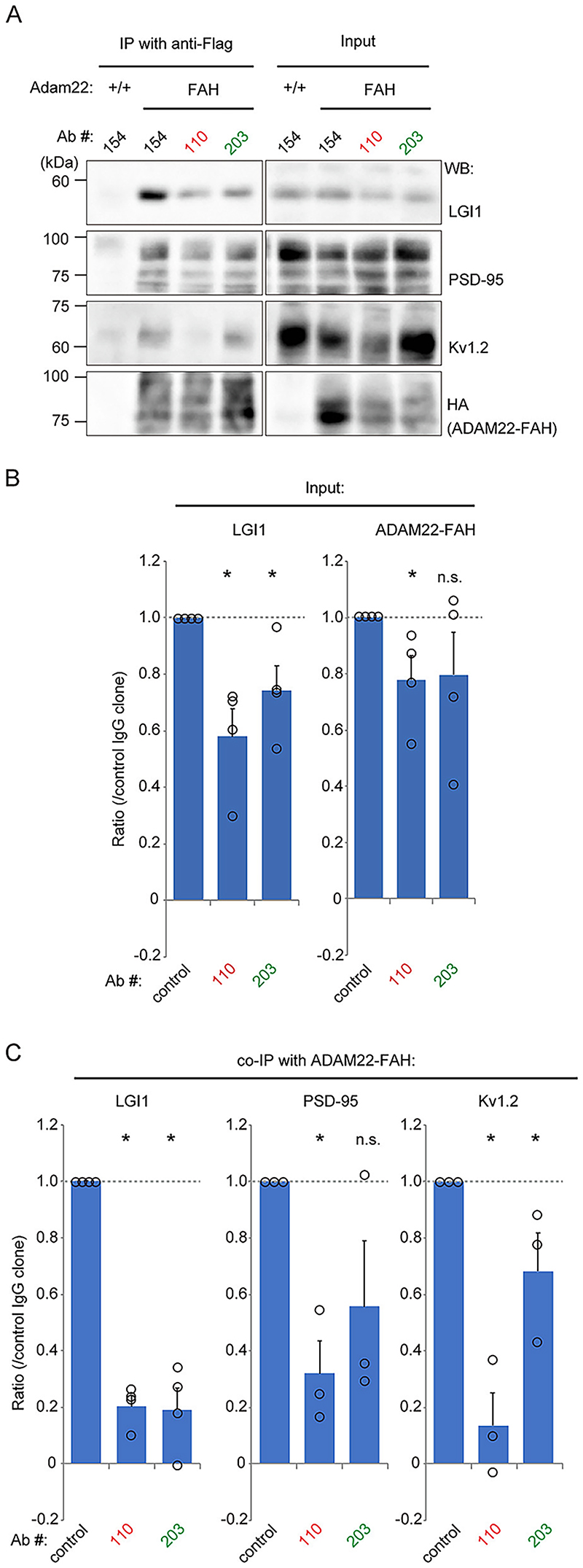
Anti-LGI1 mAbs affect the integrity of ADAM22 protein complexes in cultured mouse neurons. Treatment of cultured neurons derived from *Adam22*^FAH^ knock-in mice with anti-LGI1 mAbs (AB060-110, LRR antibody; AB060-203, EPTP antibody) significantly reduces LGI1 expression and largely reduces the functional complex including ADAM22, PSD-95 and Kv1.2. Results of ADAM22-FAH co-IP experiments are shown as (A) Western Blots, (B) quantifications of input and (C) quantifications of co-IP. Note that AB060-110 shows greater, inhibitory effect on the functional complex than AB060-203. n = 4 experiments for LGI1, n = 3 experiments for PSD-95/Kv1.2. *p < 0.05 as compared to control; Kruskal-Wallis with post-hoc Steel test. Measurements shown as mean ± SEM.

Seizure frequency *in vivo* was highest on day 2 of anti-LGI mAb infusion (Fig. 4D). To further characterize electrophysiological changes induced by anti-LGI1 mAbs at this time-point, we retrieved hippocampal brain slices from rats injected with a single ICV injection of anti-LGI1 or control mAbs after 48 h. Whole-cell patch clamp recordings were made from pyramidal cells in CA3 (Fig. 5A). There was a reduction in the frequency of spontaneous excitatory postsynaptic currents (sEPSCs) (Fig. 5B) but no change in the amplitude (Fig. 5C). Reduced sEPSCs might be attributed to the dysfunction of transsynaptic LGI1-ADAM22 complex (Fig. 6), which is required to maintain the excitatory synaptic transmission (Fukata et al., 2021; Lovero et al., 2015). Reduced excitatory synapse function in the inhibitory interneurons may cause the disinhibition of the neural network and hyperexcitability. Evaluation of the excitability of recorded cells in CA3 by injecting depolarizing currents under current clamp configuration and counting the numbers of action potentials (APs) generated demonstrated hyperexcitability in anti-LGI1 mAb-injected slices as compared to those injected with control mAbs (Fig. 5D, E) similar to that seen in the mice (Fig. 2A). There were no changes in resting membrane potential or resistance (Fig. 5F, G). These combined results highlight both direct and potential compensatory complex synaptic, cellular and circuit mechanisms that are exerted to control network excitability following anti-LGI1 mAb exposure (Joseph and Turrigiano, 2017; Kornau et al., 2020; Petit-Pedrol et al., 2018; Wright et al., 2021). Binding of the anti-LGI1 mAbs was confirmed on post-mortem immunohistochemistry (see Fig. 5H-K).

### Anti-LGI1 mAbs destabilize functional ADAM22-based complexes in primary mouse neuron cultures

To gain insight into the mode of action of the anti-LGI1 mAb used in the previous experiments (AB060-110), we treated primary cultured neurons derived from ADAM22 knock-in mice, harboring a tandem tag composed of FLAG, AU1 and HA (referred to as an FAH tag) with AB060-110 (targeting the LRR domain of LGI1), AB060-203 (targeting the EPTP domain of LGI1) or control antibodies (AB060-144 or AB060-154) for 2–3 days. We found that the expression level of LGI1 was significantly reduced in neurons treated with either AB060-110 or AB060-203, compared with control neurons (Fig. 6A, B). Furthermore, a robust reduction in LGI1 co-purified with ADAM22 was detected in neurons treated with either AB060-110 or AB060-203 (Fig. 6A, C). The interaction of ADAM22 with PSD-95 and Kv1.2 was also largely reduced by both anti-LGI1 mAbs. Interestingly, AB060-110 treatment showed an even greater inhibitory effect than AB060-203 treatment.

## 4 Discussion

Limbic encephalitis with autoantibodies to LGI1 is associated with a distinct neuro-immunological clinical phenotype characterized by facio-brachial dystonic seizures progressing to cognitive and memory impairment, focal seizures and encephalopathy (Irani et al., 2013; Thompson et al., 2018). In this study, intracerebroventricular infusion of a CSF-derived anti-LGI1 autoantibody into rodent brains caused convulsive and non-convulsive seizures mimicking the epileptic phenotype in LGI1 encephalitis patients during the acute disease phase, as well as changes in behavior and acute brain MRI scans. These results confirm and extend the findings of other studies using monoclonal anti-LGI1 antibodies derived from peripheral B cells (Ramberger et al., 2020; Upadhya et al., 2024).

Previous studies investigating the pathogenicity of patient-derived anti-LGI1 antibodies have either demonstrated memory deficits or convulsive seizures *in vivo* (Petit-Pedrol et al., 2018; Ramberger et al., 2020; Upadhya et al., 2024). LGI1 antibodies may differ in their ictogenic potential, (Ritzau-Jost et al., 2024) but the source of antibody, species of experimental animal and passive transfer techniques will all effect resulting behaviors. The anti-LGI1 mAb studied here, AB060-110, has shown a strong effect on the intrinsic excitability of CA3 neurons *in vitro* (Kornau et al., 2020). Our experiments with primary neurons indicate that AB060-110 reduces hippocampal LGI1 levels and – although this antibody did not directly compete with the LGI1-ADAM22 interaction *in vitro* (Kornau et al., 2020) – efficiently destabilizes LGI1 and ADAM22-based protein complexes. Importantly, this included a loss of PSD-95 and Kv1.2 associated with ADAM22. AB060-110 may either (i) bind to secreted, free LGI1 and the LGI1-AB060-110 complex may be less competent in binding to ADAM22 and/or (ii) induce the internalization of LGI1-ADAM22 complexes as previously reported for other LRR antibodies (Ramberger et al., 2020), both resulting in a reduced number of functional ADAM complexes. In our animal models, it is likely that AB060-110 induced similar effects, reducing the brain levels of LGI1 protein and ADAM complexes to cause seizures.

Anti-LGI1 mAb-infused mice showed an anxiety-like, hypoactive phenotype. Despite normal motor function/locomotion, reduced motility was particularly evident post-ictally, a phenotype known to be linked to epileptic seizures in mice.^33^ The reduced sociability in the three-chamber test could be attributed to altered social interaction behavior. Alongside memory impairment, commonly seen in LGI1 encephalitis (Petit-Pedrol et al., 2018; Ramberger et al., 2020), the observed behavioral abnormalities fit within the expanding spectrum of LGI1 encephalitis-associated neuropsychiatric symptoms, which include anxiety, depression, disorientation, confusion and agitation (Coleman et al., 2024; Miller et al., 2020; Zhang et al., 2024).

While brain MRI is usually unremarkable in patients with FBDS, mediotemporal lobe T2/FLAIR hyperintensities are seen in most patients in the limbic encephalitis stage, together with glucose hypermetabolism in basal ganglia and mediotemporal lobes (Heine et al., 2015). The increased hippocampal volume observed in the current rodent MRI study correlates with these findings. Later follow-up in patients with LGI1 encephalitis regularly shows hippocampal or whole brain atrophy, also in patients with formerly unremarkable MRI. LGI1 is expressed in both neuronal and glial cells in the pons, unlike its exclusive expression in neuronal cells in the hippocampus. This broader expression in the pons may increase the region’s vulnerability to cell death, potentially leading to the observed subtle shrinkage (Head et al., 2007). Future work will assess whether similar atrophy occurs long-term in rodents using the present murine models.

Continuous wireless depth EEG recordings from the CA3 region of the hippocampus revealed an initial rapid onset of convulsive seizures within 48 h of antibody infusion showing phenotypic parallels with FBDS. This was followed by a second, smaller peak of ictal activity and overall deterioration of the EEG with loss of normal background features and frequent interictal epileptiform events. This observed qualitative and quantitative EEG change reflects the natural history of LGI1 autoantibody-associated seizures observed in one of the first-described patient cohorts that presented with an increasing frequency of facio-brachial dystonic seizures until they reached a maximum at the onset of epileptic encephalopathy/limbic encephalitis (Binks et al., 2018; Irani et al., 2011). It is well established that immunotherapy can halt the progression to limbic encephalitis if FBDS is identified and treated acutely in patients (Irani et al., 2013). The current model could facilitate pre-clinical treatment trials providing a window of intervention between the first peak of seizures and normal interictal EEG and subsequent deterioration of interictal EEG to assess therapeutic effectiveness.

A recently developed pharmacological rat model for LGI1 encephalitis created by injection of DTX-K into primary motor cortex to inhibit Kv1.1 channels also revealed striking similarities to patient EEG findings and seizure semiology (Baudin et al., 2022). The human electroclinical syndrome of LGI1 encephalitis is varied, but a slow EEG wave, detected by frontal or fronto-central electrodes, has been reported in a few cases to precede facio-brachial dystonic seizures and indeed this was fully characterized in the DTX-K pharmacological model (Baudin et al., 2022; Steriade et al., 2018). However, in a recent systematic review of EEG findings in LGI1 encephalitis (combining 23 case reports and 14 case series), epileptiform discharges were the most frequent finding and most commonly arose from the temporal region (Roberto et al., 2020), as seen in this and our previously described rat model EEG recordings (Upadhya et al., 2024). The recordings were taken from region CA3 of the hippocampus that contains a particularly high density of LGI1 protein in excitatory and inhibitory synapses (Ramirez-Franco et al., 2022). *In vitro*, pathogenic and epileptogenic changes induced by human-derived LGI1 antibodies have been described in CA3 (Kornau et al., 2020; Lalic et al., 2011; Romoli et al., 2019), and in patients this area was found to be particularly sensitive to damage leading to memory deficits (Miller et al., 2020; Miller et al., 2017). The importance and frequency of seizures as a clinical symptom in the early stages of the three most prevalent forms of antibody-positive autoimmune encephalitides was highlighted in a recent study analyzing 320 patients (Kaaden et al., 2022). Seizures were present in almost two-thirds of these patients, and in the anti-LGI1 mAb-positive cases they were most likely to be focal and of temporal origin. This further validates this model as an accurate representation of the clinical and electrophysiological human LGI1 autoimmune encephalitis phenotype.

The use of continuous Video-EEG recording allowed us to accurately quantify ictal activity and categorize further into convulsive and non-convulsive seizure events. In both peaks of seizures non-convulsive seizures were more common. In a recent clinical study using systematic prolonged Video-EEG in patients with LGI1 and CASPR2 antibody encephalitis, seizures were recorded in 4 out of 20 patients that had been subjectively “seizure-free” for at least 3 months (Baumgartner et al., 2022) suggesting a proportion of patients may have persistent seizures that have not been identified. This underlines the importance of prolonged Video-EEG telemetry in these patients and validates this approach in disease models (Kreye et al., 2021b; Upadhya et al., 2024; Wright et al., 2021).

The animal models introduced here provide valuable insights into the evolution of limbic encephalitis with antibodies to LGI1 and can eventually help to understand the persisting and secondary synaptic changes that lead to progressive cognitive impairment and chronic epilepsy in some patients.

## Supplementary Material

**Appendix A. Supplementary material**

Supplementary data to this article can be found online at https://doi.org/10.1016/j.bbi.2025.02.019.

Supplementary video 1

## Data Availability

Data will be made available on request.

## Abbreviations

AIE: autoimmune encephalopathy
DTX-K: dendrotoxin-K
EEG: electroencephalographic
FBDS: faciobrachial dystonic seizures
FAH: FLAG, AU1 and HA tag
ISI: inter-spike-interval
LFP: Local field potential
anti-LGI1 mAb: monoclonal anti-LGI1 antibody
OSI: OpenSource Instruments
P21: postnatal day 21
PSBB: Post-Seizure Behavioral Battery
T2w: T2 weighted
